# Sn(SO_4_)_2_·2H_2_O from synchrotron powder data

**DOI:** 10.1107/S2414314624011799

**Published:** 2024-12-10

**Authors:** Øystein Slagtern Fjellvåg, Helmer Fjellvåg

**Affiliations:** a Institute for Energy Technology, Department Hydrogen Technology PO Box 40 NO-2027 Kjeller Norway; b Centre for Materials Science and Nanotechnology, Department of Chemistry, University of Oslo, PO Box 1033, NO-0315 Oslo, Norway; Vienna University of Technology, Austria

**Keywords:** tin(IV) sulfate dihydrate, crystal structures, Sn(IV), powder diffraction

## Abstract

Tin(IV) sulfate dihydrate, Sn(SO_4_)_2_·2H_2_O, was prepared in a reflux of sulfuric acid under oxidizing conditions. Its crystal structure was determined from powder synchrotron X-ray diffraction data and is constructed of (100) layers of [SnO_4_(H_2_O)_2_] octa­hedra (point group symmetry 1) corner-connected by sulfate tetra­hedra. Hydrogen bonds of moderate strength between the water mol­ecules and sulfate O atoms hold the layers together.

## Abstract 2

Tin(IV) sulfate dihydrate comprises of (100) layers of [SnO_4_(H_2_O)_2_] octa­hedra corner-connected by sulfate tetra­hedra, and with O—H⋯O hydrogen-bonding between the layers.

## Graphical abstract

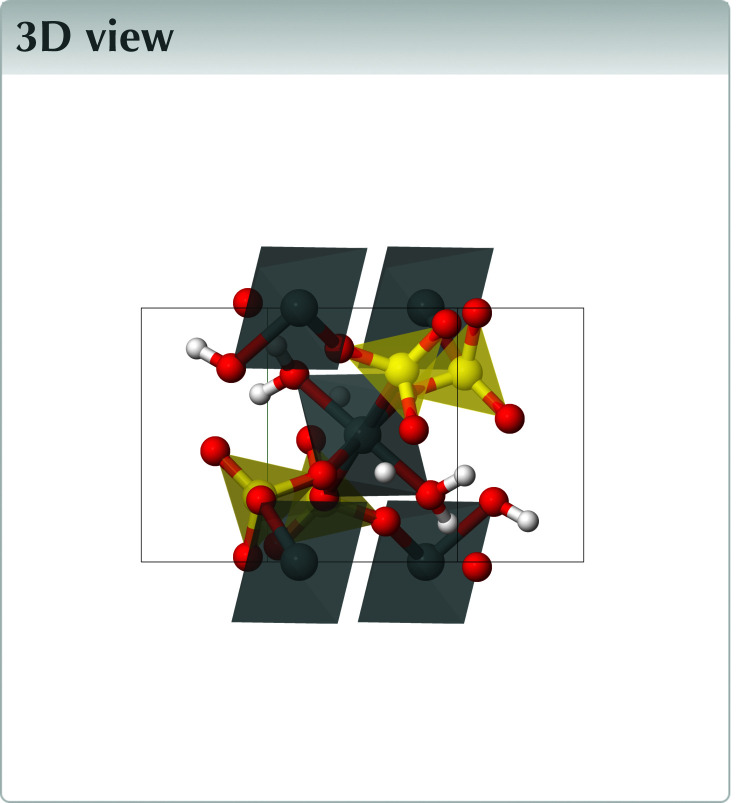

## Structure description

Tin sulfates and derivated compounds display an inter­esting structural chemistry. A number of tin sulfates, including an oxide sulfate, were reported by Ahmed *et al.* (1998 ▸) based on synthesis in 50–95%_wt_ H_2_SO_4_ at and above room temperature. In addition to the earlier reported and reasonably characterized compounds of Sn^II^SO_4_ (Rentzeperis, 1962 ▸), Sn^II^_2_OSO_4_ (Lundren *et al.*, 1982 ▸), Sn^II^_3_O(OH)_2_SO_4_ (Grimvall, 1975 ▸; Davies *et al.*, 1975 ▸), Sn^II^_6_O_4_(SO_4_)(OH)_2_ (Locock *et al.*, 2006 ▸) and Sn_7_(OH)_12_(SO_4_)_2_ (Sn^II^_6_Sn^IV^(OH)_12_(SO_4_)_2_; Grimvall, 1982 ▸), the high-temperature reactions in concentrated sulfuric acid revealed the existence of two polymorphs of Sn(SO_4_)_2_·2H_2_O (*A* and *B*), the tetra­hydrate Sn(SO_4_)_2_·4H_2_O, the mixed-valent Sn^II^/Sn^IV^ oxide sulfate Sn_6_O(SO_4_)_9_ and two polymorphs of anhydrous Sn(SO_4_)_2_, of which one is obtained on heating to around 773 K (Ahmed *et al.*, 1998 ▸). Since all the Sn^IV^-containing compounds are highly hygroscopic, handling and characterization require inert conditions. Loss of crystallinity was rapidly observed for samples subjected to air at ambient conditions. More recently, Sn^IV^(SO_4_)_2_ and mixed-valent Sn^II^Sn^IV^(SO_4_)_3_ were reported (Hämmer *et al.*, 2021 ▸). The former adopts a crystal structure with [SnO_6_] octa­hedra corner-connected through sulfate tetra­hedra in all directions, while the latter adopts a layered structure.

We report here on the crystal structure of one of the above mentioned compounds, Sn(SO_4_)_2_·2H_2_O (*B*), for which Ahmed *et al.* (1998 ▸) suggested a monoclinic structure with *a* = 9.705 (1) Å, *b* = 5.652 (1) Å, *c* = 7.033 (1) Å, *β* = 106.86 (1)^*o*^ based on powder X-ray diffraction data. On heating, Sn(SO_4_)_2_·2H_2_O (*B*) transforms into anhydrous Sn(SO_4_)_2_ at about 623 K.

**Figure 1:**
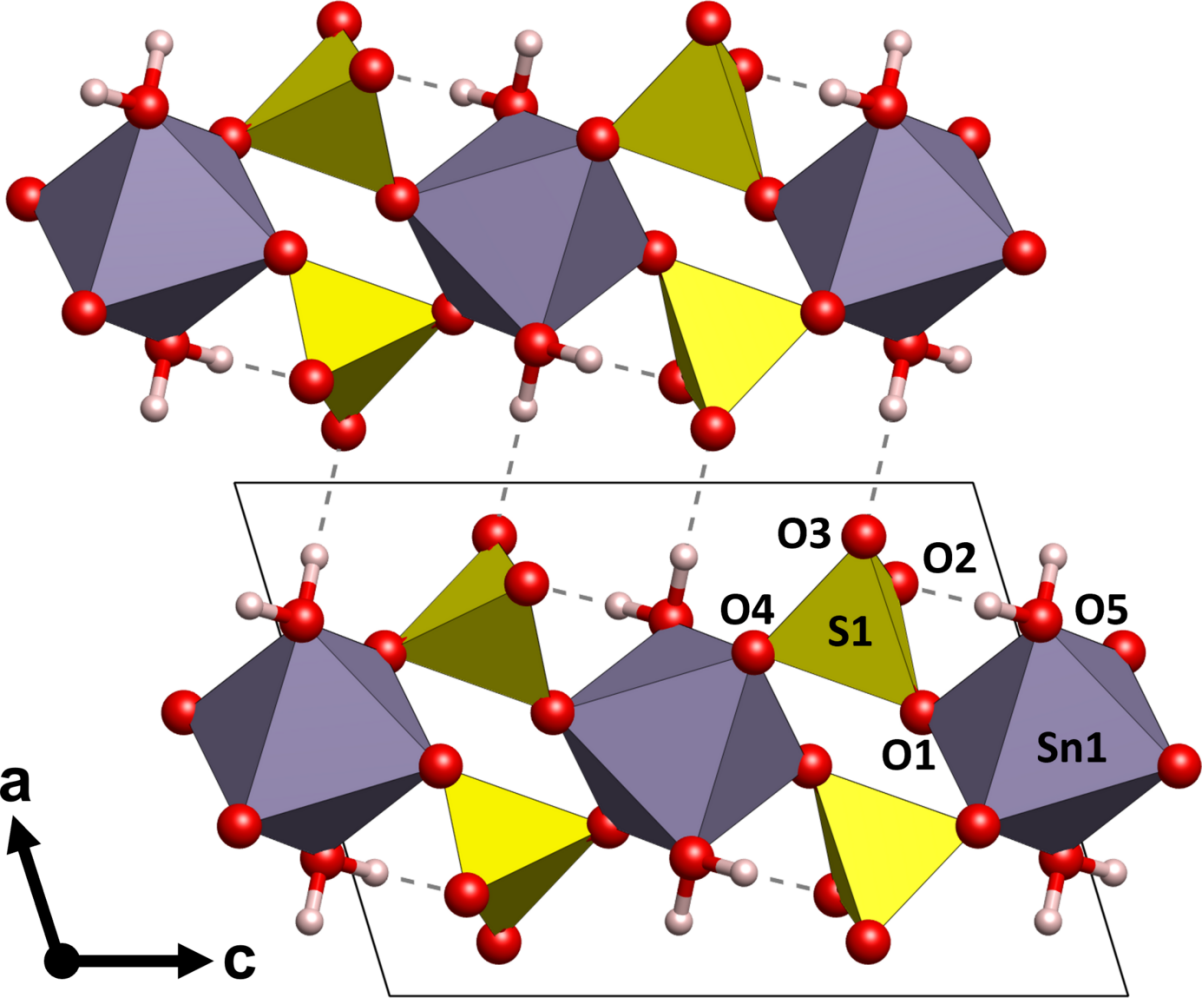
Crystal structure of Sn(SO_4_)_2_·2H_2_O in a view along [010]. Purple coordination polyhedra represent [SnO_4_(H_2_O)_2_] octa­hedra, and the yellow polyhedra the sulfate tetra­hedra. Dashed lines indicate hydrogen bonds.

The crystal structure of Sn(SO_4_)_2_·2H_2_O is shown in Figs. 1 ▸ and 2 ▸, and selected bond lengths and angles are given in Table 1 ▸. The structure can be described as being constructed of layers of slightly distorted [SnO_4_(H_2_O)_2_] octa­hedra corner-connected by sulfate tetra­hedra. There is no bonding directly between the [SnO_4_(H_2_O)_2_] units. The layers extend parallel to (100) and are stacked along [100], Fig. 1 ▸. The Sn^IV^ atom is situated at an inversion centre (multiplicity 2, Wyckoff letter *b*) and is surrounded by four sulfate groups that connect the [SnO_4_(H_2_O)_2_] units, and by two water mol­ecules. Considering the Sn—O bond lengths, we find them to be in excellent agreement for Sn^IV^ with bond lengths from 1.968 (6) to 2.046 (6) Å (Table 1 ▸). In comparison, bond lengths of 2.016 (3) – 2.049 (3) Å are observed for Sn^IV^(SO_4_)_2_ by Hämmer *et al.* (2021 ▸). The two water mol­ecules (O5) are directed towards the inter-layer space and exhibit the longest of the Sn—O bonds. The sulfate group shows a slight scatter in the S—O bond lengths, between 1.465 (6) and 1.526 (6) Å, around the ideal bond length of ∼1.49 Å (Louisnathan *et al.*, 1977 ▸). Some deviations in the lengths are expected due to the different local environments of the sulfate group as two of its oxygen atoms are directed toward Sn, while the other two are directed toward hydrogen atoms.

Intra-and inter­layer O—H⋯O hydrogen bonding between water mol­ecules and sulfate O atoms is observed. The intra­layer hydrogen bond is rather strong [O5⋯O2^ii^ = 2.541 (8) Å], whereas the inter­layer hydrogen bond, which is responsible for the cohesion of the layers along [100], is of moderate strength [O5⋯O3^ii^ = 2.735 (7) Å]. Other numerical values for these inter­actions are compiled in Table 2 ▸.

## Synthesis and crystallization

The current sample of Sn(SO_4_)_2_·2H_2_O (*B*) was obtained according to Ahmed *et al.* (1998 ▸), by reacting Sn powder (Fluka; 99.9%) in 85%_wt_ H_2_SO_4_ at 368 K in reflux while oxygen gas was passed through the reaction mixture. The obtained product was isolated after ten days by deca­nting, followed by washing and drying before storage in a vacuum desiccator.

## Refinement

**Figure 3:**
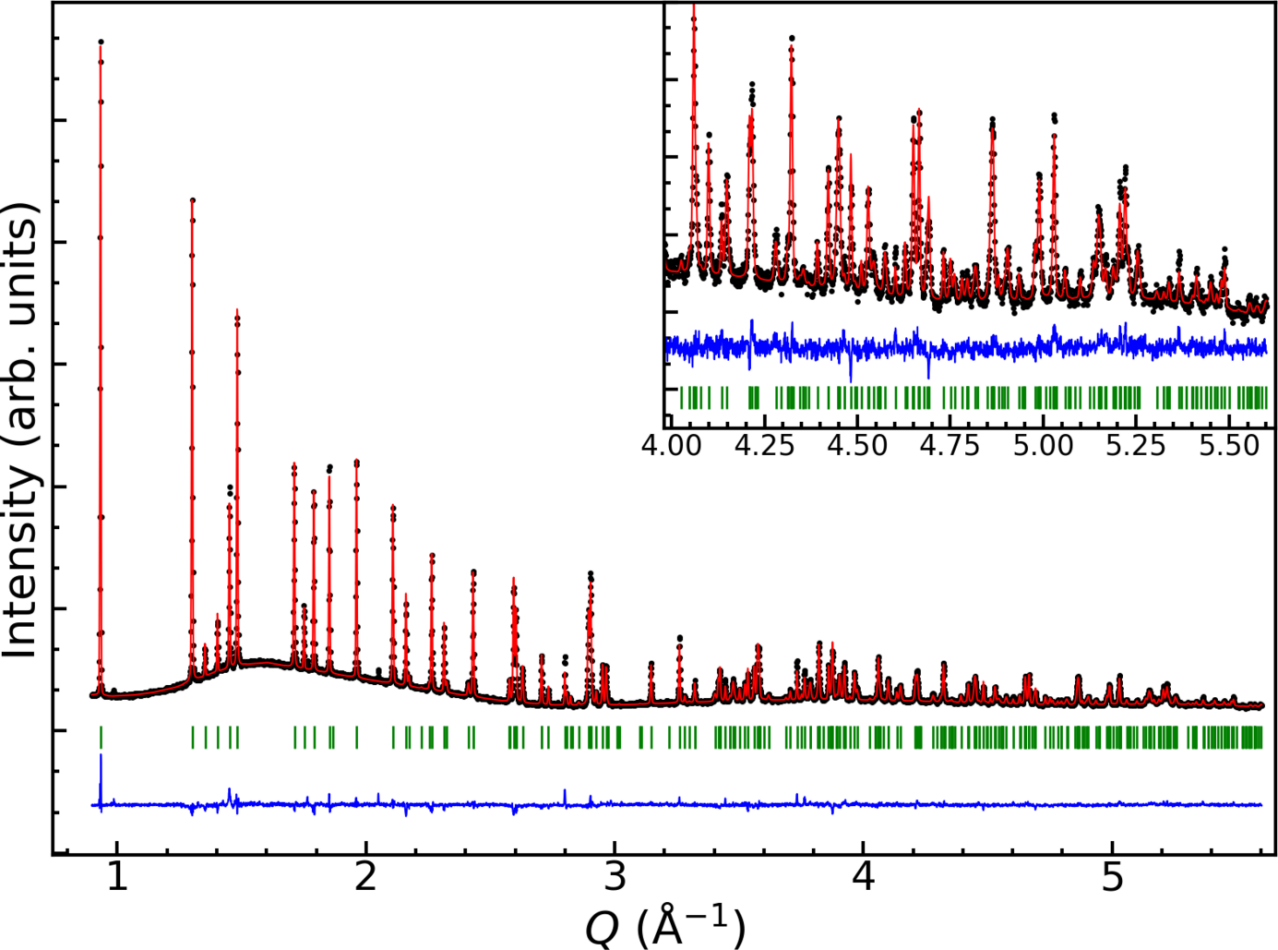
Observed (black), calculated (red), and difference (blue) intensity profile from Rietveld refinement of synchrotron X-ray data for Sn(SO_4_)_2_·2H_2_O. Bragg reflections are marked with green bars.

Crystal data, data collection and structure refinement details are summarized in Table 3 ▸. Synchrotron X-ray data of an Sn(SO_4_)_2_·2H_2_O powder sample was collected in a 0.5 mm capillary at BM01B at the Swiss–Norwegian beamlines (SNBL), the European Synchrotron Radiation Facility (ESRF), Grenoble, France, with a wavelength of 1.00098 Å using a high-resolution detector. The data revealed the Sn(SO_4_)_2_·2H_2_O sample to be of high purity, with two minor impurity reflections visible at about 1 and 2.05 Å^−1^ (Fig. 3 ▸). The indexed cell given by Ahmed *et al.* (1998 ▸) was used as a starting point for the structure solution. For the final refinement, the *a* and *c* axes were inter­changed relative to the setting used by Ahmed *et al.* (1998 ▸). Le Bail refinements with the reported lattice parameters yielded a good fit, and space group *P*2_1_/*c* as the space group. Charge flipping using *SUPERFLIP* (Palatinus & Chapuis, 2007 ▸) implemented in *JANA2006* (Petříček *et al.*, 2014) quickly gave a good model for the atomic positions for the heavier elements. Without the constraint for the S—O bond length, unrealistically large variations were obtained. The ideal bond length for the S—O bond in a sulfate group is ∼1.49 Å (Louisnathan *et al.*, 1977 ▸). The crystal structure was refined with distance restraints on the S—O bond length by selecting the target distance to 1.49 Å, a value of 0.002 Å for allowed deviations, and a moderate penalty factor.

Based on a previous report regarding composition and charge neutrality (Ahmed *et al.*, 1998 ▸), we added hydrogen atoms to the structure. They were placed to have an O—H bond length of ∼0.95 Å and with an angle of ∼105° between the H atoms. The H atoms were further directed towards O2 and O3 as the lengths to these oxygen atoms indicate hydrogen bonding (Table 2 ▸). Displacement parameters were not refined for hydrogen atoms. Rietveld refinement of the final structural model is shown in Fig. 3 ▸. The refinement included lattice parameters, pseudo-Voigt peak shape, background, zero error, axial divergence correction, axial strain broadening tensors, atomic parameters, and thermal displacement parameters of non-H atoms.

## Supplementary Material

Crystal structure: contains datablock(s) global, I. DOI: 10.1107/S2414314624011799/wm4223sup1.cif

CCDC reference: 2407647

Additional supporting information:  crystallographic information; 3D view; checkCIF report

## Figures and Tables

**Figure 2:**
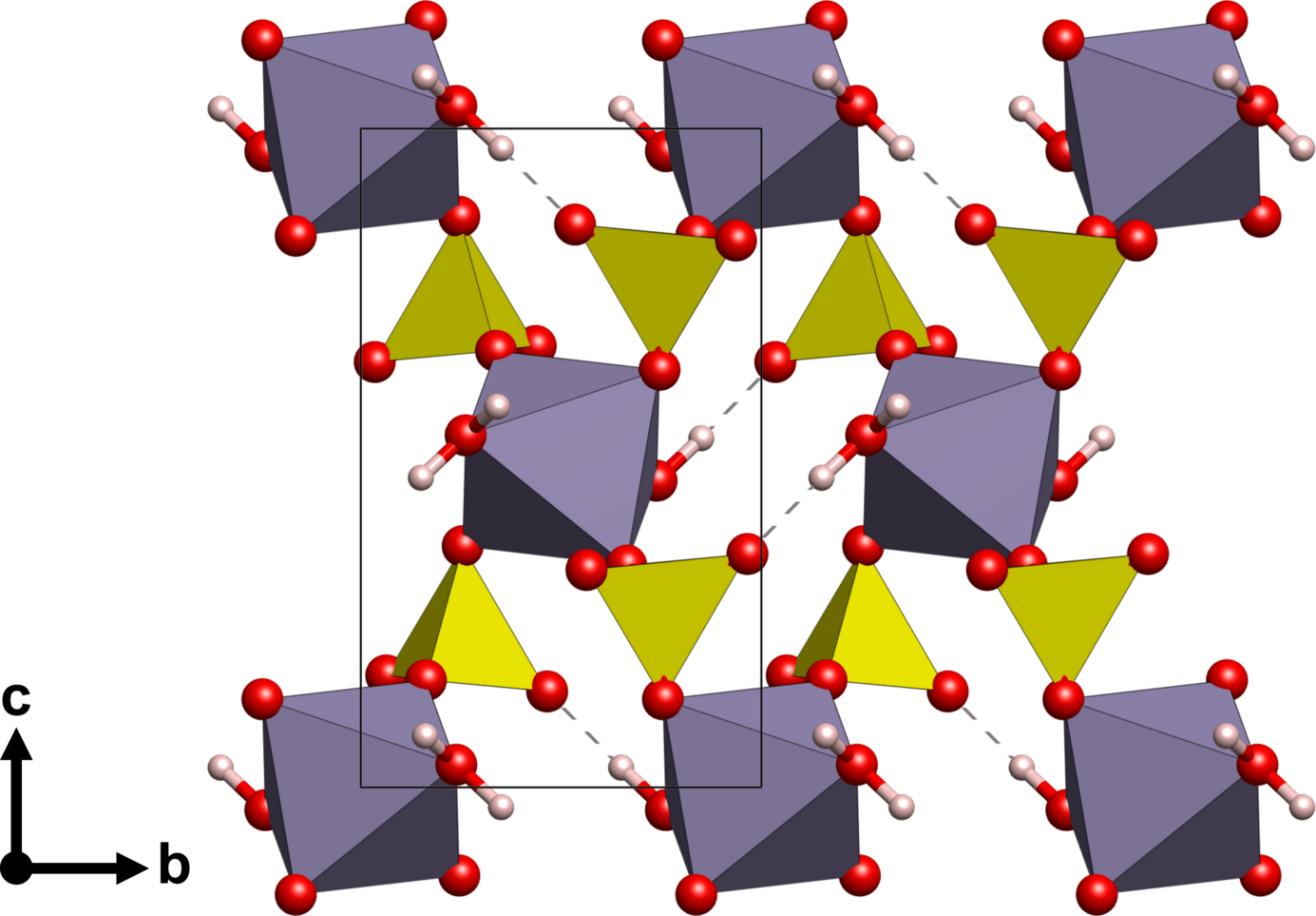
Crystal structure of Sn(SO_4_)_2_·2H_2_O in a view along [100]. Colour code is as in Fig. 1 ▸.

**Table 1:** Selected geometric parameters (Å, °)

Sn1—O1	1.968 (6)	S1—O2	1.465 (6)
Sn1—O4^i^	2.042 (5)	S1—O3	1.467 (6)
Sn1—O5	2.046 (6)	S1—O4	1.495 (5)
S1—O1	1.526 (6)		
			
H1—O5—H2	103.2 (6)		

Symmetry code: (i) 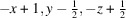.

**Table 2:** Hydrogen-bond geometry (Å, °)

*D*—H⋯*A*	*D*—H	H⋯*A*	*D*⋯*A*	*D*—H⋯*A*
O5—H1⋯O2^ii^	0.969 (6)	1.574 (5)	2.541 (8)	175.6 (4)
O5—H2⋯O3^iii^	0.963 (6)	1.780 (4)	2.735 (7)	171.0 (4)

Symmetry codes: (ii) 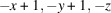; (iii) 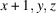.

**Table 3:** Experimental details

Crystal data	
Chemical formula	Sn(SO_4_)_2_·2H_2_O
*M*_r_	346.86
Crystal system, space group	Monoclinic, *P*2_1_/*c*
Temperature (K)	293
*a*, *b*, *c* (Å)	7.03475 (5), 5.65165 (5), 9.70464 (7)
β (°)	106.8524 (4)
*V* (Å^3^)	369.27 (1)
*Z*	2
Radiation type	Synchrotron, λ = 1.00098 Å
μ (mm^−1^)	10.46
Specimen shape, size (mm)	Cylinder, 40 × 0.5

Data collection	
Diffractometer	High-resolution sychrotron
Specimen mounting	Capillary
Data collection mode	Transmission
Scan method	Step
2θ values (°)	2θ_min_ = 8.207 2θ_max_ = 53, 2θ_step_ = 0.007

Refinement	
*R* factors and goodness of fit	*R*_p_ = 0.024, *R*_wp_ = 0.033, *R*_exp_ = 0.017, *R*(*F*) = 0.045, χ^2^ = 3.792
No. of parameters	46
No. of restraints	4
H-atom treatment	H-atom parameters constrained

Computer programs: local program, *JANA2006 and *JANA2020** (Petříček *et al.*, 2014 ▸), *SUPERFLIP* (Palatinus & Chapuis, 2007 ▸), *VESTA* (Momma & Izumi, 2011 ▸) and *publCIF* (Westrip, 2010 ▸).

## full crystallographic data

### Tin(IV) sulfate dihydrate Crystal data

**Table:**

Sn(SO_4_)_2_·2H_2_O	*Z* = 2
*M**_r_* = 346.86	*F*(000) = 332
Monoclinic, *P*2_1_/*c*	*D*_x_ = 3.120 Mg m^−^^3^
Hall symbol: -P 2ycb	Synchrotron radiation, λ = 1.00098 Å
*a* = 7.03475 (5) Å	µ = 10.46 mm^−^^1^
*b* = 5.65165 (5) Å	*T* = 293 K
*c* = 9.70464 (7) Å	White
β = 106.8524 (4)°	cylinder, 40 × 0.5 mm
*V* = 369.27 (1) Å^3^	Specimen preparation: Prepared at 368 K

### Tin(IV) sulfate dihydrate Data collection

**Table:**

High-resolution sychrotron diffractometer	Absorption correction: for a cylinder mounted on the φ axis
Specimen mounting: Capillary	
Data collection mode: transmission	2θ_min_ = 8.207°, 2θ_max_ = 53°, 2θ_step_ = 0.007°
Scan method: step	

### Tin(IV) sulfate dihydrate Refinement

**Table:**

*R*_p_ = 0.024	4 restraints
*R*_wp_ = 0.033	8 constraints
*R*_exp_ = 0.017	H-atom parameters constrained
*R*(*F*) = 0.045	Weighting scheme based on measured s.u.'s
6400 data points	(Δ/σ)_max_ = 0.044
Profile function: Pseudo-Voigt	Background function: Manual background combined with 5 Legendre polynoms
46 parameters	Preferred orientation correction: none

### Tin(IV) sulfate dihydrate Fractional atomic coordinates and isotropic or equivalent isotropic displacement parameters (Å^2^)

**Table:**

	*x*	*y*	*z*	*U*_iso_*/*U*_eq_	
Sn1	0.5	0	0	0.0331 (3)*	
S1	0.2572 (4)	0.2445 (5)	0.2045 (3)	0.0339 (8)*	
O1	0.4385 (6)	0.1591 (8)	0.1624 (5)	0.0244 (18)*	
O2	0.1956 (6)	0.4801 (8)	0.1454 (4)	0.0324 (16)*	
O3	0.1010 (6)	0.0638 (8)	0.1670 (5)	0.043 (2)*	
O4	0.3254 (7)	0.2572 (8)	0.3653 (4)	0.0187 (17)*	
O5	0.7294 (8)	0.2365 (9)	0.0353 (6)	0.0414 (19)*	
H1	0.76	0.35	−0.03	0.01*	
H2	0.855	0.16	0.078	0.01*	

### Tin(IV) sulfate dihydrate Geometric parameters (Å, º)

**Table:**

Sn1—O1	1.968 (6)	S1—O2	1.465 (6)
Sn1—O4^i^	2.042 (5)	S1—O3	1.467 (6)
Sn1—O5	2.046 (6)	S1—O4	1.495 (5)
S1—O1	1.526 (6)		
			
H1—O5—H2	103.2 (6)	O1—S1—O3	109.7 (3)
O1^ii^—Sn1—O4^i^	88.53 (18)	O1—S1—O4	104.7 (3)
O1^ii^—Sn1—O4^iii^	91.47 (18)	O2—S1—O3	114.9 (3)
O1^ii^—Sn1—O5	95.0 (2)	O2—S1—O4	109.5 (3)
O1^ii^—Sn1—O5^ii^	85.0 (2)	O3—S1—O4	106.5 (3)
O1—S1—O2	110.9 (3)		

Symmetry codes: (i) −*x*+1, *y*−1/2, −*z*+1/2; (ii) −*x*+1, −*y*, −*z*; (iii) *x*, −*y*+1/2, *z*−1/2.

### Tin(IV) sulfate dihydrate Hydrogen-bond geometry (Å, º)

**Table:**

*D*—H···*A*	*D*—H	H···*A*	*D*···*A*	*D*—H···*A*
O5—H1···O2^iv^	0.969 (6)	1.574 (5)	2.541 (8)	175.6 (4)
O5—H2···O3^v^	0.963 (6)	1.780 (4)	2.735 (7)	171.0 (4)

Symmetry codes: (iv) −*x*+1, −*y*+1, −*z*; (v) *x*+1, *y*, *z*.
